# Supplementary material for: Prevalence and Associated Factors of Compliance Behaviors among Middle-Aged and Older Hypertensive Patients in China: Results from the China Health and Retirement Longitudinal Study
Source: Int J Environ Res Public Health. 2020 Oct 8;17(19):7341. doi: 10.3390/ijerph17197341 (PMC7579574; doi:10.3390/ijerph17197341)
Supplement: Supplementary file 1 [file ijerph-17-07341-s001.pdf]

Supplementary Table S1. The source, re-coding, and definition of the research variables.

Variables	Corresponding Items in CHARLS 2015 Questionnaire	Definition or Categorization
Hypertension	**DA007.** Have you been diagnosed with [conditions listed below, read one by one] by a doctor ?<br>1. Hypertension<br>**DA008.** Do you know if you have *hypertension* [preload the current choice in DA007]?<br>1. Yes; 2. No; 3. Don't know	Respondents chose "1. Hypertension" in **DA007** and/or "1. Yes" in **DA008** were identified as hypertensive patients.
Compliance behaviors		
Compliance with medication	**DA013.** Are you now taking any of the following treatments to treat or control your hypertension? (Check all that apply) Taking Chinese traditional medicine, taking Western modern medicine?<br>1. Taking Chinese traditional medicine<br>2. Taking Western modern medicine<br>3. None of the above	Participants who answered 1 or 2 were considered as compliance with medication.
Compliance with blood pressure monitoring	**DA012.** During last year (last 12 months), how many times have you had blood pressure examination?<br>_____0…999 Times	Participants who reported ≥12 times/year were considered as compliance with blood pressure monitoring.
Predictor variables (Downstream)		
Age	**BA002.** What is your actual date of birth?	45–54; 55–64; 65–74; ≥75
Gender	**BA000_W2_3.** Interviewer record R's gender.<br>1. Male; 2. Female	Male; Female
BMI		Underweight (BMI <18.5 kg/m^2);<br>Normal (18.5 kg/m^2 ≤ BMI < 24 kg/m^2);<br>Overweight (24 kg/m^2 ≤ BMI < 28 kg/m^2);<br>Obese (BMI ≥28 kg/m^2)
Number of complications	**DA007.** Have you been diagnosed with [conditions listed below, read one by one] by a doctor?<br>1. Hypertension<br>2. Dyslipidemia (elevation of low-density lipoprotein, triglycerides (TGs), band total cholesterol, or a low high-density lipoprotein level)<br>3. Diabetes or high blood sugar<br>4. Cancer or malignant tumor (excluding minor skin cancers)<br>5. Chronic lung diseases, such as chronic bronchitis, emphysema (excluding tumors, or cancer)<br>6. Liver disease (except fatty liver, tumors, and cancer)<br>7. Heart attack, coronary heart disease, angina, congestive heart failure, or other heart problems<br>8. Stroke<br>9. Kidney disease (except for tumor or cancer)<br>10. Stomach or other digestive disease (except for tumor or cancer)<br>11. Emotional, nervous, or psychiatric problems<br>12. Memory-related disease	0; 1; 2; and ≥3

	13. Arthritis or rheumatism<br>14. Asthma	
ADL	**Basic Activities of Daily Living, BADL**<br>**DB014.** Because of health and memory problems, do you have any difficulties with using the toilet, including getting up and down?<br>**DB012.** Because of health and memory problems, do you have any difficulty with eating, such as cutting up your food ? (Definition: By eating, we mean eating food by oneself when it is ready)<br>**DB010.** Because of health and memory problems, do you have any difficulty with dressing? Dressing includes taking clothes out from a closet, putting them on, buttoning up, and fastening a belt.<br>**DB015.** Because of health and memory problems, do you have any difficulties with controlling urination and defecation? If you use a catheter (conduit) or a pouch by yourself, then you are not considered to have difficulties.<br>**DB013.** Do you have any difficulty with getting into or out of bed?<br>**DB011.** Because of health and memory problems, do you have any difficulty with bathing or showering?<br>**Instrumental Activities of Daily Living, IADL**<br>**DB016.** Because of health and memory problems, do you have any difficulties with doing household chores? (Definition: By doing household chores, we mean house cleaning, doing dishes, making the bed, and arranging the house)<br>**DB017.** Because of health and memory problems, do you have any difficulties with preparing hot meals? (Definition: By preparing hot meals, we mean preparing ingredients, cooking, and serving food)<br>**DB018.** Because of health and memory problems, do you have any difficulties with shopping for groceries? By shopping, we mean deciding what to buy and paying for it.<br>**DB035.** Because of health and memory problems, do you have any difficulties with making phone calls?<br>**DB020.** Because of health and memory problems, do you have any difficulties with taking medications? By taking medications, we mean taking the right portion of medication right on time.<br>**DB019.** Because of health and memory problems, do you have any difficulties with managing your money, such as paying your bills, keeping track of expenses, or managing assets?<br>For each question above, five options are provided:<br>1. No, I do not have any difficulty<br>2. I have difficulty but can still do it<br>3. Yes, I have difficulty and need help<br>4. I cannot do it	The answer of "No, I don't have any difficulty" refers to normal ability of this activity, otherwise means impaired ability. Participants who reported impaired function in any one of the 12 activates were considered as ADL decline.
Predictor variables (Midstream)		
Smoking	**DA059.** Have you ever chewed tobacco, smoked a pipe, smoked self-rolled cigarettes, or smoked cigarettes/cigars?<br>1. Yes; 2. No	Answer "yes" refers to smoking.
Drinking	**DA067.** Did you drink any alcoholic beverages, such as beer, wine, or liquor in the past year? How often?	Answer 1 or 2 refers to current drinking.

	1. Drink more than once a month<br>2. Drink but less than once a month<br>3. None of these	
Exercising	**DA051.** During a usual week, did you do any […] for at least 10 min continuously? (yes or no)<br>A. Now, think about all the vigorous activities requiring hard/high intensity physical effort that you do in a usual week. Vigorous activities make you breathe much harder than normal and may include heavy lifting, digging, plowing, aerobics, fast bicycling, and cycling with a heavy load. Think only about those physical activities that you did for at least 10 min at a time.<br>B. Now think about activities which take moderate physical effort that you do in a usual week. Moderate physical activities make you breathe somewhat harder than normal and may include carrying light loads, bicycling at a regular pace, or mopping the floor. Again, think about only those physical activities that you did for at least 10 min at a time.<br>C. Now think about the time you spend walking in a usual week. This includes at work and at home, walking to travel from place to place, and any other walking that you might do solely for recreation, sport, exercise, or leisure.<br>**DA052.** During a usual week, on how many days did you do […] for at least 10 min?<br>_____1…7 days	Participants who exercised 5–7 d/week for more than 10 min/d were considered as regular exercise.
Sleep duration	**DA049.** During the past month, how many hours of actual sleep did you get at night (average hours for one night)? (This may be shorter than the number of hours you spend in bed.)<br>_____0…999 h	<6 h; 6–8 h; ≥9 h
Depression	**DC009-DC018,** 10-Item Center for Epidemiologic Studies Depression Scale, CESD-10	The total score of CESD-10 ≥10 were considered as having depressed mood.
Cognitive function	**Orientation:**<br>**DC001.** Please tell me today's date.<br>1. Year is correct; 2. Month is correct; 3. Day is correct<br>**DC002.** Please tell me the day of the week.<br>1. Day of week is correct; 2. Day of week is incorrect<br>**DC003.** What is the current season<br>1. Season is correct; 2. Season is incorrect<br>**Attention:**<br>**DC019.** Let's try some subtraction of numbers this time. What does 100 minus 7 equal? _____<br>**DC020.** And 7 from that?_____<br>**DC021.** And 7 from that?_____<br>**DC022.** And 7 from that?_____<br>**DC023.** And 7 from that?_____<br>**Memory:**<br>**DC027.** A little while ago, I read you a list of words and you repeated the ones you could remember. Please tell me any of the words that you remember now.<br>**Visual spatial ability:**<br>**DC025.** Do you see this picture? Please draw that picture on this paper.<br>1. Drew the picture; 2. Failed to draw the picture	Cognitive function was measured through orientation, attention, memory, and visual spatial ability, in which the total score ranged from 0 to 21. Participants' cognitive function as "scored <10" and "scored ≥10".

Predictor variables (Upstream)		
Marital status	**BE001** R' Marital Status: What is your marital status?<br>1. Married with spouse present<br>2. Married but not living with spouse temporarily for reasons such as work<br>3. Separated<br>4. Divorced<br>5. Widowed<br>6. Never married<br>7. Cohabitated	Married (answer 1 or 2);<br>Non-married (answer 3, 4, 5, 6, 7)
Place of residence	**BB000_W3_2.** Was it village or city/town?<br>1. Main city zone<br>2. Combination zone between urban and rural areas<br>3. The town center<br>4. ZhenXiang area<br>5. Special area<br>6. Township central<br>7. Village	Urban (answer 1, 2, 3, 4, or 5)<br>Rural (answer 6 or 7)
Educational level	**BD001_W2_4.** Has your highest level of education changed from last wave? If so, what is the highest level of education you have attained now? (not including adult education)<br>1. No formal education (illiterate)<br>2. Did not finish primary school<br>3. Sishu/home school<br>4. Elementary school<br>5. Middle school<br>6. High school<br>7. Vocational school<br>8. Two-/Three-Year College/Associate degree<br>9. Four-Year College/Bachelor's degree<br>10. Master's degree<br>11. Doctoral degree/Ph.D.	No formal education or illiterate (answer 1, 2, or 3);<br>Educational level = 6years (answer 4);<br>Educational level = 9years (answer 5);<br>Educational level ≥12years (answer 6, 7, 8, 9, 10, or 11).
Personal income	**GA001.** Did you receive any wage and bonus income in the past year?<br>1. Yes; 2. No<br>**GA003.** Did you receive any of the following types of individual income in the past year? (check all that apply)<br>1. Unemployment compensation<br>2. Pension subsidy for the oldest old/pension voucher<br>3. Workers' compensation from Industrial Accident Compensation Insurance including wage-replacement benefits, disability benefits, and survivors' benefits<br>4. Elderly family planning subsidies<br>5. Medical aid<br>6. Other government subsidies	Participants who answer "No" in **GA001** and "None of the above" in **GA003** were considered as having no personal income, otherwise, as having personal income.

	7. Social assistance<br>8. Other income sources<br>9. None of the above	
Medical insurance	**EA001.** Are you the policy holder/primary beneficiary of any of the types of health insurance listed below? (circle all that apply)<br>1. Urban employee medical insurance (yi-bao)<br>2. Urban resident medical insurance<br>3. New cooperative medical insurance (he-zuo-yi-liao)<br>4. Urban and rural resident medical insurance<br>5. Government medical insurance (gong-fei)<br>6. Medical aid<br>7. Private medical insurance: purchased by work unit<br>8. Private medical insurance: purchased by individual<br>9. Urban non-employed persons' health insurance<br>10. Other medical insurance (specify)____<br>11. No insurance	Participants who answer "No insurance" were considered as have no medical insurance, otherwise, have medical insurance.
Health education	**DA013**. Have your care providers ever given you health education/advice on the following (check all that apply)? Weight control, exercise, diet and/or smoking control?<br>1. Weight control; 2. Exercise; 3. Diet; 4. Smoking control; 5. None of the above	When participants answer, "None of the above", it refers to not having received health education; otherwise, it means having received health education.
